# Characterization and Biological Evaluation of Propolis from Poland

**DOI:** 10.3390/molecules22071159

**Published:** 2017-07-11

**Authors:** Milena Popova, Efstathia Giannopoulou, Krystyna Skalicka-Woźniak, Konstantia Graikou, Jaroslaw Widelski, Vassya Bankova, Haralabos Kalofonos, Gregory Sivolapenko, Katarzyna Gaweł-Bęben, Beata Antosiewicz, Ioanna Chinou

**Affiliations:** 1 Institute of Organic Chemistry with Centre of Phytochemistry, Bulgarian Academy of Sciences, 1113 Sofia, Bulgaria; popova@orgchm.bas.bg (M.P.); bankova@orgchm.bas.bg (V.B.); 2 Division of Pharmacognosy and Chemistry of Natural Products, Departments of Pharmacy, National and Kapodistrian University of Athens, 15771 Athens, Greece; kgraikou@pharm.uoa.gr; 3 Clinical Oncology Laboratory, University Hospital of Patras, Patras Medical School, 26504 Rio, Greece; egiannopoulou@zeincro.com (E.G.); kalofonos@upatras.gr (H.K.); 4 Department of Pharmacognosy with Medicinal Plant Unit, Medical University of Lublin, 20093 Lublin, Poland; jwidelski@pharmacognosy.org; 5 Pharmacokinetics Laboratory, Department of Pharmacy, University of Patras, 26504 Rio, Greece; gsivolap@upatras.gr; 6 Department of Cosmetology, University of Information Technology and Management in Rzeszow, 35225 Rzeszów, Poland; kagawel@wsiz.rzeszow.pl (K.G.-B.); bantosiewicz@wsiz.rzeszow.pl (B.A.)

**Keywords:** GC–MS, cytotoxic activity, Polish propolis, *Populus nigra*, *Populus tremula*

## Abstract

In this study, we assessed the therapeutic potential of propolis from Poland and performed chemical analysis by GC–MS, as well as determined its botanical origin. Chemical constituents typical for bud exudates of *Populus nigra* (section *Aigeiros*) were determined, however, glycerol esters of phenolic acids, as well as unusually high amounts of *p*-coumaric and ferulic acid and their benzyl esters, were also detected. These constituents are characteristic for buds of *Populus tremula* (section *Leuce*). We also evaluated the antiproliferative effect of propolis extracts against nine human cancer cell lines. Additionally, promising antibacterial activity of the dichloromethane extract (Minimal Inhibitory Concentration MIC values of 0.95–1.24 mg/mL), as well as a moderate antifungal activity (MIC values of 1.25–1.40 mg/mL), was noticed. Propolis from Poland appeared as a rich source of antibacterial and antiproliferative compounds and this confirmed that it is a valuable natural product with the potential to improve human health.

## 1. Introduction

Propolis, a natural resinous substance collected by honey bees from buds and exudates of plants, is thought to be used in the beehive as a protective barrier against the bees’ enemies. It has been used in folk medicine for centuries, for the treatment of wounds, burns and stomach ulcers, and for other therapeutic applications. Nowadays, it has been shown that propolis possesses antimicrobial, antiseptic, anti-inflammatory and antitumor activities [1,2,3,4], and it is extensively used in food, beverages and food supplements to improve health and prevent diseases [5].

The chemical composition of propolis depends strongly on the plant sources available to the bees in different geographical and climatic regions. In the temperate zone, bud exudates of *Populus* species belonging to the section *Aigeiros* are the main propolis plant sources [5]. These exudates contain flavonoids and aromatic acid esters, which are responsible for most of its biological and pharmacological activities [1,6]. Generally, it is very important to evaluate the flavonoidal content in foods, as there has been an increasing interest due to the growing evidence of their health benefits through epidemiological studies [7]. Knowledge of the chemical composition and biological properties of propolis from different regions is extremely valuable with respect to the problem of different propolis types, as well as with their standardization [8].

In the existing publications on Polish propolis, the geographic origin of all studied samples is not described. They report the chemical analyses of selected samples, with quantification of the main phenolic acids [9] and the isolation of selected flavonoids [10], while their antibacterial activity against only the gram-positive *Staphylococcus aureus* [9,11] has been evaluated. In the study frame of Eurasian propolis, a few Polish samples were analyzed, and *Populus nigra*, together with trembling aspen and birch, were identified as their plant sources [12,13]. In two other publications, the cytostatic and antitumor activity of different, unspecified propolis fractions has been studied [14,15], without any further chemical analysis. Moreover, previous studies of Kubina et al. [4] identified in an ethanolic extract of polish propolis, collected in southern Poland, a high amount of phenolic acids, and Polish propolis samples in five strains were tested. Additionally, in the same study, anti-proliferative influence on selected neoplastic cells was demonstrated.

In this study, we report on the antibacterial, antifungal and antiproliferative activities of propolis from Poland, accompanied by its chemical analysis by the GC–MS method after the microscopic pollen determination of the tested sample.

## 2. Results and Discussion

### 2.1. Propolis Composition

The chemical composition of the studied propolis sample collected in Poland (dichloromethane and methanol extracts) was investigated by GC–MS after silylation. According to the GC–MS analysis, the methanol extract contained mainly sugars (>50%), and it showed weak antibacterial and antiproliferative activity, while it appeared absolutely inactive against all assayed fungi. For this reason, only the results for the chemical composition of the dichloromethane extract have been tabled (Table 1).

Eighty-five constituents were identified. The investigated sample showed a chemical profile of propolis originating from bud exudates of *Populus* species: its main constituents were flavonoids, phenolic acids and their esters (Table 1). Among them, the flavonoids (pinocembrin, pinobanksin and its 3-*O*-acetate, chrysin, and galangin) and phenolic acid esters (penthenyl and phenylethyl caffeates) are typical constituents for the bud exudates of *P. nigra* section *Aigeiros* [16], which was the main plant source of the studied sample. Previously, ten flavonoids, mainly flavones, were reported in Polish propolis [10]; among these were tectochrysin, genkwanin, apigenin, pilloin, and 5-hydroxy-4′,7-dimethoxyflavone, but they were not detected in our sample. Instead, flavanones and dihydroflavonols were the most abundant constituents in our study. On the other hand, a series of esters of *p*-coumaric acid and ferulic acid were detected, and the percentage of benzyl esters, as well as of *p*-coumaric and ferulic acids, was unusually high (>17%). The presence of significant amounts of these compounds, as well as the presence of the glycerol esters of phenolic acids (also found earlier in Polish propolis samples), was very characteristic for the bud of *Populus tremula* section *Leuce* [12,13,17].

A high amount of phenolic acids such as caffeic, gallic, ferulic, and coumaric acids, as well as benzoic acid, were identified in ethanolic extract of Polish propolis collected in southern Poland abundant in alder (*Alnus glutinosa*), horsechestnut (*Aesculus hippocastanum*), black poplar (*P. nigra*), beech (*Fagus sylvatica*) and birch (*Betula alba*) [4].

### 2.2. Antimicrobial Activity

The two extracts of the studied propolis from Poland were evaluated for their antimicrobial activity against six gram-negative and -positive bacterial strains, and three human pathogenic fungi. The results of these tests (Table 2) showed interesting and promising antibacterial activity of the dichloromethane extract (MIC values of 0.90–1.34 mg/mL), as well as a moderate antifungal activity (MIC values of 1.25–1.40 mg/mL). Previous studies of Kubina et al. [4] evoked activity of Polish propolis samples in a range of 0.39–6.25 mg/mL, but only five strains were tested, and no reference substance was used. Another study on an ethanolic extract of Polish propolis displayed varying effectiveness against 12 methicillin-sensitive and -resistant *Staphylococcus aureus* with a MIC within the range of 0.39 to 0.78 mg/mL [18].

The differences in the antibacterial activity of propolis extracts depend on the collection region and the races of the honey bees. Some studies revealed that propolis exerts synergistic effects with antibiotics, acting on the bacterial wall structure and ribosome function. The mechanism of propolis antibacterial activity seems to be linked to some of its components. The potent bacteriostatic and bactericidal effects of propolis can be associated with their combined action, manifested by an inhibition of protein synthesis and bacterial growth by preventing cell division [18,19]. The activity could be attributed mainly to the high content of flavonoids such as galangin, pinocembrin and pinobanksin, which are known to possess high antimicrobial (antibacterial as well as fungicidal) activity [3]. Galangin and caffeic acids are enzymatic inhibition agents responsible for the inhibition of bacterial growth and proliferation. In addition, some active substances composing propolis may disorganize the cytoplasmic membrane and cell wall, with the effect of a partial bacteriolysis. Flavonoids affect the bacterial membrane potential and cause permeability alteration within the inner microorganism membrane [18,20].

### 2.3. Antiproliferative Activity

Propolis extracts were tested for antiproliferative activity in several cell lines 48 h after their application in cells, and the results were compared to the antitumor effect of doxorubicin and farmorubicin [21,22]. Both MTT and LDH (lactate dehydrogenase) assays were used for evaluating the effect of propolis extractions. The MTT assay was used to determine the metabolically active viable cells. The LDH assay was used for detecting cytotoxicity caused by external effects (i.e., the addition of an agent). The principle of this assay is based on the measurement of LDH release after the disruption of the plasma membrane resulting in cell death. The two methods reflect different time points of cell death, as the decrease of the cell metabolism appears at the initial steps of cell death, and membrane damage occurs in the advanced stage of cell death [23]. Although an appropriate approach for comparing the activity of propolis extracts among the different types of cancer cells or among the standard chemotherapeutic reagents is the extrapolation of IC_50_ values, this could not be applied in the current study because the available data points were too few to derive such values with confidence. Further studies should be carried out in order for a more exact characterization of the activity of propolis extracts in terms of the IC_50_ values to be obtained.

Previous studies [4] of an ethanolic extract of propolis (EEP) from southern Poland by the MTT assay showed a cytotoxic effect towards the Me45 cell line of malignant melanoma and the HCT 116 (Human Colorectal Carcinoma) cell line of colorectal cancer in a range of 7.36 to 25.76% (concentrations tested: 3.125–100 µg/mL). Both growth and a cell size reduction were observed. According to Szliszka et al. [13] the percentage of apoptotic cells after exposure to 50 μg/mL EEP from Polish propolis increased to 71.10% on tumor necrosis factor-related apoptosis-inducing ligand (TRIAL)-resistant HeLa cells. Apigenin and caffeic acid phenylether ester (CAPE) may be compounds responsible for this activity. Synergistic induction of many other cancer cell lines of TRIALs with either propolis extract or single isolated phenolic compounds was noticed [24].

### 2.4. The Effect of Propolis Extracts in Lung Cancer Cell Proliferation and Toxicity

**Figure 1:**
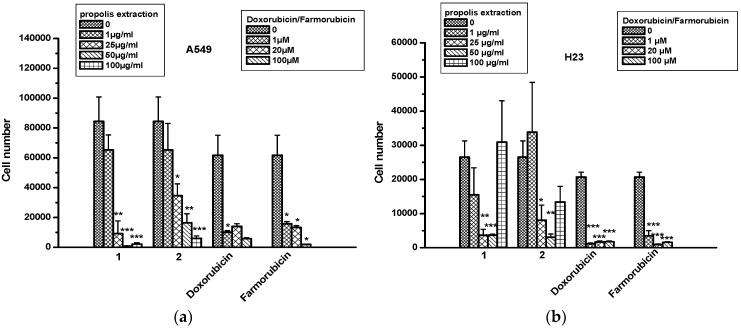
The effect of propolis extracts 1 and 2 on the proliferation of non-small cell lung cancer (NSCLC) cells. (**a**) A549 and (**b**) H23 cells. Asterisks denote statistically significant differences compared to untreated cells. * *p* < 0.05, ** *p* < 0.001 and *** *p* < 0.0001.

Propolis extracts were tested for antitumor effects in human non-small cell lung cancer (NSCLC) cell lines A549 and H23 (Figure 1). It was found that both propolis extracts decreased A549 cell numbers in a dose-dependent manner. Propolis extract 1 (dichloromethane extract) showed a better antitumor effect at the doses of 50 and 100 μg/mL than doxorubicin and farmorubicin, and no toxic effect was found for either extract 1 or 2 by measuring the secreted LDH amounts in supernatants. In H23 cells, both propolis extracts decreased cell numbers in a dose-dependent manner, however, this effect was reversed at the maximum tested dose of 100 μg/mL. This is not the first time that a compound exerted a biphasic response. In the literature, numerous agents have showed such a behavior, known as a hormetic mechanism [25]. Furthermore, extract 1 showed a toxic effect only in H23 cells, which was not observed for extract 2, compared to doxorubicin and farmorubicin (Table 3). The toxic effect was evaluated according to LDH levels; the greater the levels of LDH, the greater the toxicity for the tested extracts.

### 2.5. The Effect of Propolis Extracts in Colon Cancer Cell Proliferation and Toxicity

**Figure 2:**
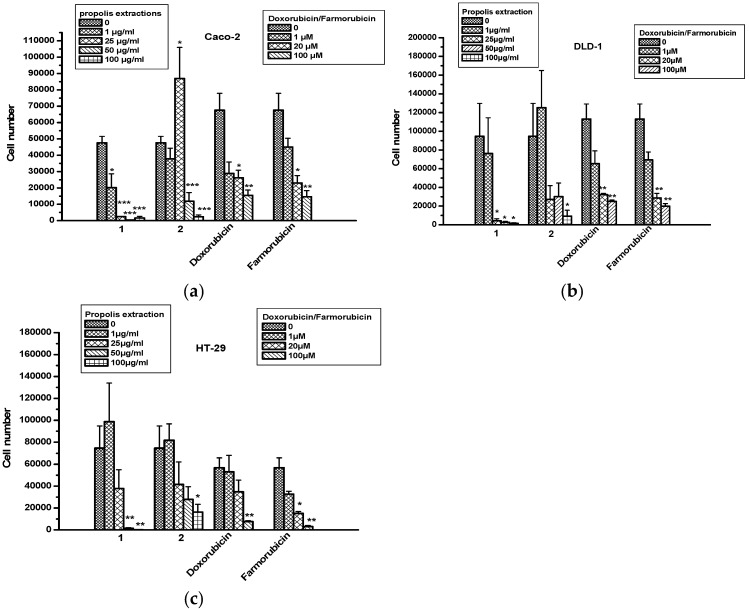
The effect of propolis extracts 1 and 2 on the proliferation of colon cancer cells. (**a**) Caco-2; (**b**) DLD-1 and (**c**) HT-29. Asterisks denote statistically significant differences compared to untreated cells. * *p* < 0.05, ** *p* < 0.001 and *** *p* < 0.0001.

Propolis extracts were tested for cytotoxic/cytostatic effects in colon cancer cell lines Caco-2, DLD-1 and HT-29. Extract 1 exhibited a strong inhibitory effect for all tested doses in Caco-2 cells, compared to extract 2, as well as doxorubicin and farmorubicin (Figure 2a–c). The decrease in colon cancer cell numbers caused by extract 1 was in line with an increase in cytotoxicity according to the measurement of LDH amounts in supernatants (Table 3). No toxicity was measured in any colon cancer cell lines with extract 2.

### 2.6. The Effect of Propolis Extracts in Breast Cancer Cell Proliferation and Toxicity

**Figure 3:**
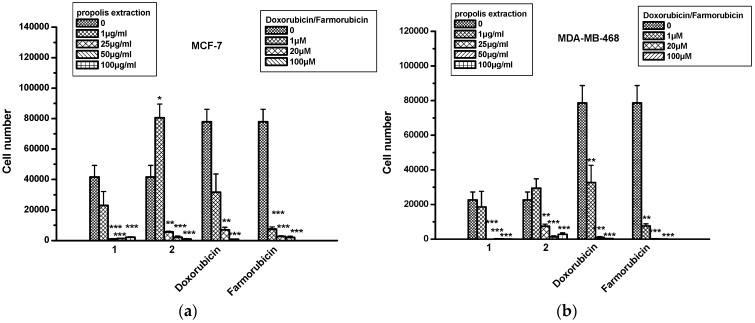
The effect of propolis extracts 1 and 2 on the proliferation of breast cancer cells. (**a**) MCF-7 and (**b**) MDA-MB-468 cells. Asterisks denote statistically significant differences compared to untreated cells. * *p* < 0.05, ** *p* < 0.001 and *** *p* < 0.0001.

Propolis extracts were tested in human breast cancer cell lines MCF-7 (hormone-dependent) and MDA-MB-468. We found that both extracts decreased MCF-7 and MDA-MB-468 cell numbers in a dose-dependent manner for concentrations higher than 1 μg/mL (Figure 3). Extracts 1 and 2 had equivalent effects to doxorubicin and farmorubicin when they were used in concentrations equal to or higher than 1 μM (Figure 3). No cytotoxicity was observed, according to the LDH assay, for any of the extracts, compared to doxorubicin and farmorubicin (Table 4). It is worth emphasizing that CAPE, a well-known active compound from bee propolis also detected in our extracts, has been previously identified as a strong antioxidant, anti-inflammatory, antiviral and anticancer molecule. Its influence on inducing cell death by inhibiting NFκB and by inducing pro-apoptotic pathways is well documented. CAPE, as well as its structural analogues, has been found [26] to induce breast cancer apoptosis in p53-mutated cell lines.

### 2.7. The Effect of Propolis Extracts in Glioblastoma Cell Proliferation and Toxicity

**Figure 4:**
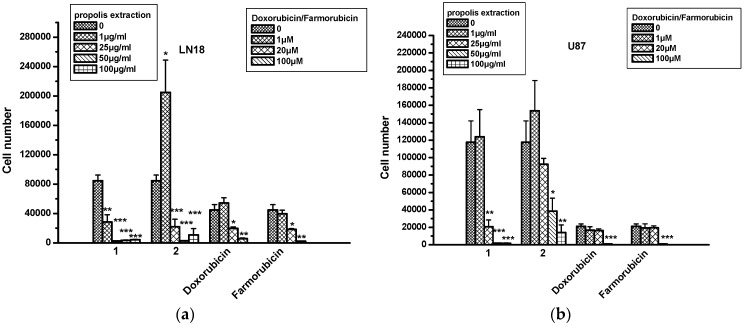
The effect of propolis extracts 1 and 2 on the proliferation of glioblastoma cells. (**a**) LN18 and (**b**) U87 cells. Asterisks denote statistically significant differences compared to untreated cells. * *p* < 0.05, ** *p* < 0.001 and *** *p* < 0.0001.

LN18 and U87 cell lines were used for testing the cytotoxic/cytostatic activity of propolis extracts. It was found that extract 1 decreased LN18 cell numbers in a dose-dependent manner for all tested concentrations (Figure 4a), while extract 2 was also active for concentrations higher than 1 μM. Surprisingly, we noticed that propolis extracts were superior to doxorubicin and farmorubicin when they were used in concentrations equal to or higher than 25 μg/mL. In U87 cells, extract 1 was superior to extract 2 for all tested doses (Figure 4b). No cytotoxicity was found for extracts 1 and 2 in either cell line, in contrast to for doxorubicin and farmorubicin (Table 4).

### 2.8. The Effect of Propolis Extracts in Fibroblast Cell Proliferation and Toxicity

**Figure 5:**
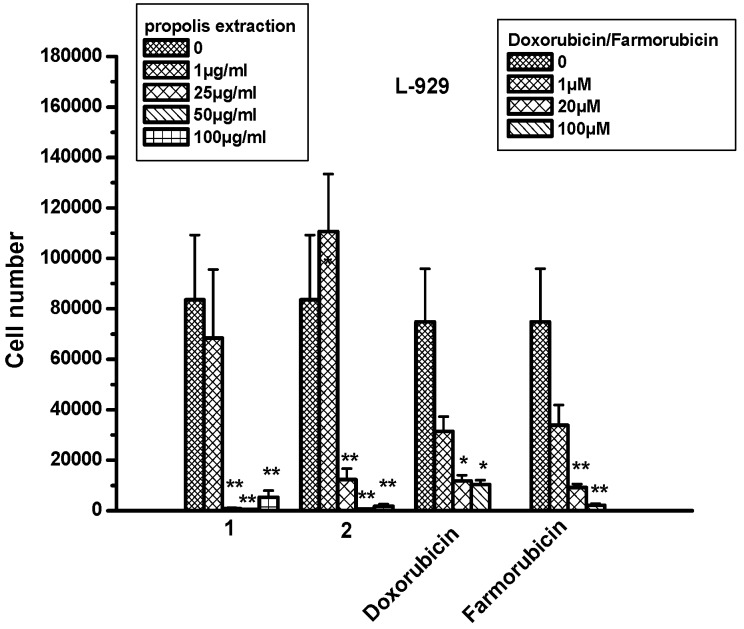
The effect of propolis extracts 1 and 2 on the proliferation of L-929 cell line. Asterisks denote statistically significant differences compared to untreated cells. * *p* < 0.05 and ** *p* < 0.001.

L-929 cells, normal mouse fibroblast cell lines, were used for testing the effect of propolis extracts in a non-cancerous cell line. The results showed that both extracts did not affect cell numbers when they were applied on cells at the concentration of 1 μg/mL. However, when higher concentrations were used, a statistically significant decrease in cell numbers was observed, similar to for doxorubicin and farmorubicin (Figure 5; Table 4).

In numerous in vitro studies, propolis exhibited proapoptotic activities on various types of neoplastic cells, such as in the cases of laryngeal cancer, lung carcinoma, pancreatic cancer, thyroid neoplasm, colorectal cancer, breast cancer, prostate cancer and malignant glioma [4]. The compounds responsible for this activity were mainly flavonoids (quercetin, apigenin, galangin, pinocembrin, and pinostrobin), which act as antioxidants. Together with CAPE, they exhibited potent anti-proliferative and proapoptotic properties. Currently, CAPE is one of the most promising compounds [4,27,28].

## 3. Materials and Methods 

### 3.1. Samples

Propolis was collected in early autumn, 2009, in the region of Nałęczów (South Poland, 20 km from Lublin; 22°12′55.054′′ E; 51°17′8.412′′ N) and concerning its pollen analysis, the main plants found were: *Populus alba*, *P. nigra, P. tremula*, *Betula verucosa*, *Acer pseudoplatanus*, *Pinus silvestris* and *Aesculus hippocastanum*. Identification was performed by Dr. Michał Hajnos from the Department of Pharmacognosy, Medical University of Lublin, Poland. The voucher specimen (PL_2009) was deposited in the Departments of Pharmacy, National and Kapodistrian University of Athens, Greece.

### 3.2. Extraction and Sample Derivatization

Propolis, grated after cooling, was extracted exhaustively with dichloromethane (extract 1; 24 h) and methanol (extract 2; 24 h; 1:10 *w*/*v*) at room temperature, and the extracts were evaporated to dryness [2]. For the silylation of the extracts, about 5 mg of the residue was mixed with 50 µL of dry pyridine and 75 µL of bis(trimethylsilyl)trifluoracetamide, heated at 80 °C for 20 min, and analyzed by GC–MS [2].

### 3.3. GC–MS Analysis

The GC–MS analysis was performed with a Hewlett Packard Gas Chromatograph 5890 Series II Plus linked to a Hewlett Packard 5972 mass spectrometer system equipped with a 23 m long, 0.25 mm id, 0.5 µm film thickness HP5-MS capillary column. The temperature was programmed from 100 to 300 °C at a rate of 5 °C/min. Helium was used as a carrier gas, with a flow rate of 0.7 mL/min. The split ratio was 1:20; injector temperature, 280 °C; and ionization voltage, 70 eV. The identification was accomplished using computer searches on NIST98 and Wiley mass spectral databases. Reference compounds previously isolated were co-chromatographed when possible, to confirm GC retention times. The components of the propolis extract were determined by considering their areas as a percentage of the total ion current.

### 3.4. Antimicrobial Bioassay

In vitro antibacterial studies, firstly, were carried out by the disc diffusion method [29] measuring the zone of inhibitions against the two gram-positive bacteria: *Staphylococcus aureus* (ATCC 25923) and *S. epidermidis* (ATCC 12228); and the four gram-negative bacteria: *Escherichia coli* (ATCC 25922), *Enterobacter cloacae* (ATCC 13047), *Klebsiella pneumoniae* (ATCC 13883) and *Pseudomonas aeruginosa* (ATCC 227853); as well as the pathogen fungi *Candida albicans* (ATCC 10231), *C. tropicalis* (ATCC 13801) and *C. glabrata* (ATCC 28838). The MIC values were determined using the dilution method in 96-well plates [29]. Standard antibiotic netilmicin (at concentrations of 4–88 μg/mL) was used, in order to control the sensitivity of the tested bacteria, while 5-flucytosine and itraconazole (at concentrations of 0.5–25 μg/mL) were used as controls against the tested fungi (Sanofi, Diagnostics Pasteur at concentrations of 30, 15 and 10 μg/mL). For each experiment, any pure solvent used was also applied as a blind control. The experiments in all cases were repeated three times, and the results were expressed as average values.

### 3.5. Antiproliferative Activity

Human colon cancer cell lines Caco-2, DLD-1 and HT-29; human NSCLC cell lines H23 and A549; human breast cancer cell lines MCF-7 and MDA-MB-468; human glioma cell lines LN18 and U87; and mouse fibroblast L-929 cell line were purchased from the American Type Culture Collection (ATCC). Caco-2, MCF-7 and L-929 cells were cultured in Eagle’s Minimum Essential Medium (EMEM) with Earle’s BSS (Balanced Salt Solution) and 2 mM l-glutamine, and supplemented with 1.0 mM sodium pyruvate, 0.1 mM nonessential amino acids, and 1.5 g/L sodium bicarbonate. Further MCF-7 cells were supplemented with 0.01 mg/mL bovine insulin. DLD-1, HT-29 and MDA-MB-468 cells were cultured in RPMI (Roswell Park Memorial Institute) 1640 medium. All media were supplemented with amphoterecin B (2.5 μg/mL), penicillin (100 units/mL), streptomycin (100 μg/mL), and gentamicin (50 μg/mL). Cells were cultured at 37 °C, 5% CO_2_ and 100% humidity. 

The extracts of propolis were diluted in DMSO with 2% ethanol, and the final concentrations in cell cultures were 1, 25, 50 and 100 μg/mL. Doxorubicin and farmorubicin, commercially available drugs, were used at concentrations of 1, 10 and 100 μM.

To determine whether the extracts of propolis affected the proliferation of cells, the MTT assay was used, as previously described [30]. Briefly, cells were seeded at a density of 1.5 × 104 cells/well in 48-well tissue culture plates. After 24 h, the medium was replaced with 0.25 ml of serum-free medium in each well, and various concentrations of propolis extracts were applied onto the cells. The number of cells was measured at 48 h. MTT stock solution (5 mg/mL in PBS (Phospate-Buffered Saline)) at a volume equal to 1/10 of the medium was added, and the plates were incubated at 37 °C for 2 h. The medium was removed, the cells were washed with PBS (pH 7.4), and 100 μL of acidified isopropanol (0.33 mL of HCl in 100 mL of isopropanol) was added to each well and agitated thoroughly in order to solubilize the dark blue formazan crystals. The samples were transferred to 96-well plates and were immediately read on a microplate reader (Tecan, Sunrise, Magellan 2) at a wavelength of 540 nm with a reference of 620 nm. A standard curve was applied for converting the O.D. (Optical Density) results to cell numbers following a standard procedure. Briefly, multiple pre-defined cell numbers were calculated using a Neubauer chamber, and the cells were seeded in 48-well tissue culture plates. After the cells were attached to the wells, MTT stock solution was added, as described above. The standard curve was plotted using the O.D. results and the groups with a known number of cells. Each cell-number count was taken in triplicate. The MTT assay is a measure for cellular dehydrogenase activity reflecting cell viability. Therefore, transforming the measured absorbance data in this way did not represent an exact count, but rather, an estimate of cell numbers.

The amount of LDH released by the cells was determined using an LDH activity kit assay (LDH kit, Roche, Germany) according to the manufacturer’s instructions, as previously described [31]. Briefly, the cells were seeded at a density of 1.5 × 104 cells/well in 48-well tissue culture plates. At 24 h, the medium was replaced with 0.25 mL of serum-free medium. Seventy-two hours after incubation, supernatants were collected and centrifuged at 250× *g* for 10 min. Supernatants of 100 μL/well transferred into 96-well plates and 100 μL/well of a reaction mixture was added (freshly prepared). After 30 min, the incubation samples were measured on a microplate reader (Tecan, Sunrise, Magellan 2) at a wavelength of 492 nm with a reference wavelength of 620 nm.

### 3.6. Statistical Analysis

Differences between groups and controls were tested by an unpaired *t*-test. Each experiment included at least triplicate measurements. All results were expressed as means ± SEM from at least three independent experiments.

## 4. Conclusions

Concluding, the investigated propolis sample contained mainly flavanones and dihydroflavonols, as well as series of esters of *p*-coumaric acid and ferulic acid. The dichloromethane extract showed an interesting profile, with a wide broad spectrum of antibacterial activity against both gram-positive and -negative bacteria, and, regarding antiproliferative activity, showed that it exerted an antitumor activity against various cancer cell lines. Although the intensity of the antiproliferative effect of propolis differed among the different cell lines, the importance of such an effect remains because it underlines a wide-range action for propolis. Cancer is not a single disease, but a spectrum of diseases with high heterogeneity, and the generic antiproliferative effect of propolis may indicate a unique type of mechanism. Furthermore, its effect in L-929 normal fibroblast cells was non-toxic at the concentration of 1 μg/mL. Moreover, it could be proposed, especially for the dichloromethane extract, to be further investigated by in vitro and/or in vivo experiments against other selected cancer cells. It will also be very important to further clarify the hormetic response of propolis, and how this might be manipulated in a way that humans will benefit from it. The present results demonstrate that propolis from Poland may be a rich source of antibacterial and antiproliferative compounds, while it is also a valuable natural product with the potential to improve human health. Therefore, propolis could be introduced as a food supplement, which could be easily up-taken through a normal diet, to ameliorate well-being. Careful consideration, through further scientific research, should be given in the future, in order to enable its use as a valuable nutraceutical. 

## Figures and Tables

**Table 1:** Chemical composition of dichloromethane propolis extract (GC–MS data).

Chemical Category	Compounds	% Total Ion Current ^a^
**Aromatic Acids**	Benzoic acid	3.1
	Dihydrocinnamic acid	0.1
	Cinnamic acid	0.1
	*p*-Hydroxybenzoic acid	0.1
	Vanillic acid	0.1
	*p*-Coumaric acid (*Z*)	0.3
	*p*-Methoxycinnamic acid	0.1
	Ferulic acid (*Z*)	0.1
	*p*-Coumaric acid (*E*)	5.0
	Dimethoxycinnamic acid	0.7
	Isoferulic acid	0.4
	Ferulic acid (*E*)	3.4
	Caffeic acid	0.1
**Other Aromatics**	Benzyl alcohol	0.3
	Phenylethyl alcohol	0.1
	*p*-Hydroxybenzaldehyde	0.1
	Hydroquinone	0.1
	Vanilin	1.0
**Fatty Acids**	Palmitic acid	0.4
	Linoleic acid	0.7
	Oleic acid	0.3
**Esters**	Benzyl benzoate	0.2
	Propyl *p*-coumarate	0.1
	Pentyl *p*-coumarate	0.2
	Butyl *p*-coumarate	0.8
	Butenyl *p*-coumarate	0.4
	Benzyl cinnamate	0.2
	Pentyl *p*-coumarate	2.6
	Pentenyl *p*-coumarate	3.4
	Isobutyl caffeate	0.1
	Pentenyl ferulate	0.1
	Benzyl *p*-coumarate (*Z*)	0.3
	2-Methylbutyl caffeate	0.1
	3-Methyl-3-butenyl caffeate	0.5
	3-Methyl-2-butenyl ferulate	0.2
	2-Methyl-2-butenyl caffeate	0.3
	3-Methyl-2-butenyl caffeate	0.5
	Phenylethyl *p*-coumarate	0.1
	Benzyl ferulate	0.1
	Benzyl *p*-coumarate (*E*)	6.7
	Coniferyl benzoate	0.6
	Benzyl ferulate	1.9
	Benzyl caffeate	1.5
	Phenylethyl caffeate	0.8
	Cinnamyl *p*-coumarate	4.0
	Cinnamyl ferulate	0.2
	Cinnamyl caffeate	0.7
	Glycerol ester of phenolic acids	0.6
	Coumaryl coumarate	0.2
**Flavonoids and Chalcones**	2′,6′-Dihydroxy-4′-methoxydihydrochalcone	1.3
	Trihydroxymonomethoxy chalcone (*m*/*z*: 502)	0.2
	Pinostrobin chalcone	1.2
	Pinocembrin chalcone	2.6
	Pinocembrin *	4.8
	Pinobanksin chalcone	0.3
	Sakuranetin	0.3
	Pinobanksin *	5.7
	3-Acetylpinobanksin chalcone	0.2
	Trihydroxyflavanone (*m*/*z*: 488)	0.2
	2′,6′-Dihydroxy-4,4′-dimethoxydihydrochalcone	0.3
	3-Acetylalpinon	0.3
	Dihydroxymethoxyflavanone (*m*/*z*: 430)	0.5
	3-Acetylpinobanksin	3.3
	2′,6′,4-Trihydroxy-4′-methoxydihydrochalcone	0.5
	Chrysin *	3.0
	Galangin *	4.0
	Dihydroxydimethoxychalcone (*m*/*z*: 444)	0.7
	Dimethoxyhydroxyflavanone (*m*/*z*: 372)	0.5
	Isosakuranetin chalcone	1.9
	Isosakuranetin	2.0
	Alpinon chalcone	2.0
	Alpinon	1.9
	Naringenin	0.2
	Kaempferol-dimethyl ether	1.3
	Kaempferol-methyl-ether	2.4
	Dihydroxymethoxyflavone (*m*/*z*: 428)	0.6
	Kaempferol *	0.3
	Quercetin-methyl-ether	0.7
**Others**	Ethylamine	0.4
	Glycerol	0.1

^a^ The ion current generated depends on the characteristics of the compound concerned and is not a true quantification. * Compounds unambiguously identified by direct comparison with authentic reference samples. All other compounds were tentatively identified by comparison of their MS data with databases.

**Table 2:** Antimicrobial activities (zones of inhibition/MIC in mg/mL; *n* = 3) of the two studied extracts (1-dichloromethane and 2-methanolic) of propolis from Poland.

Tested Compounds	*S. aureus*	*S. epidermidis*	*P. aeruginosa*	*E. cloacae*	*K. pneumoniae*	*E. coli*	*C. albicans*	*C. tropicalis*	*C. glabrata*
Propolis extr1	18/0.95	19/0.90	16/1.10	16/1.15	17/1.03	14/1.34	12/1.40	13/1.28	14/1.25
Propolis extr2	13	12	10	10	9	8	NA	NA	NA
Netilmicin	21/0.004	25/0.004	20/0.088	23/0.008	22/0.008	24/0.010	NT	NT	NT
Itraconazole	NT	NT	NT	NT	NT	NT	20/0.01	22/0.001	23/0.0001
5-flucytosine	NT	NT	NT	NT	NT	NT	21/0.01	22/0.001	24/0.0001

NT: not tested; NA: not active.

**Table 3:** The effect of propolis extracts 1 and 2 on LDH levels in A549, H23, Caco-2, DLD-1 and HT-29. Doxorubicin and farmorubicin were used as positive controls. Results are expressed as percentages compared to untreated cells.

		A549	H23	Caco-2	DLD-1	HT-29
**Propolis Extr1**	1 μg/mL	0	0	0	0	0
	25 μg/mL	59% ± 11	69% ± 1	96% ± 0.5	82% ± 2	35% ± 33
	50 μg/mL	36% ± 34	78% ± 7	112% ± 60	49% ± 38	71% ± 14
	100 μg/mL	23% ± 62	28% ± 7	67% ± 4	29% ± 55	60% ± 18
**Propolis Extr2**	1 μg/mL	5% ± 18	0	0	0	0
	25 μg/mL	19% ± 9	27% ± 1	0	74% ± 35	39% ± 29
	50 μg/mL	48% ± 50	82% ± 37	5% ± 93	72% ± 55	40% ± 48
	100 μg/mL	84% ± 60	68% ± 54	23% ± 103	110% ± 72	60% ± 57
**Doxorubicin**	20 μM	0.3% ± 0.3	1.2% ± 1.2	0.3% ± 0.3	1.2% ± 1.2	0
	100 μM	22% ± 7	21% ± 6	22% ± 7	21% ± 6	17% ± 5
**Farmorubicin**	20 μM	0	9% ± 9	0	9% ± 9	0
	100 μM	33% ± 18	28% ± 6	33% ± 18	28% ± 6	30% ± 8

**Table 4:** The effect of propolis extracts 1 and 2 on LDH levels in MCF-7, MDA-MB-468, U87, LN18 and L-929 cells. Doxorubicin and farmorubicin were used as positive controls. Results are expressed as percentages compared to untreated cells.

		MCF-7	MDA-MB-468	U87	LN18	L-929
**Propolis extr1**	1 μg/mL	0	0	0	0	0
	25 μg/mL	196% ± 183	57% ± 30	9% ± 0.5	50% ± 38	112% ± 58
	50 μg/mL	170% ± 148	42% ± 23	50% ± 43	25% ± 3	95% ± 58
	100 μg/mL	178% ± 154	40% ± 24	52% ± 42	52% ± 16	172% ± 14
**Propolis extr2**	1 μg/mL	6% ± 1	0	38% ± 39	15% ± 17	16% ± 33
	25 μg/mL	65% ± 57	0	7% ± 6	86% ± 85	202% ± 67
	50 μg/mL	87% ± 72	13% ± 55	14% ± 3	64% ± 32	221% ± 7
	100 μg/mL	146% ± 118	0	17% ± 0.5	92% ± 10	269% ± 62
**Doxorubicin**	20 μM	121% ± 2	60% ± 38	0	121% ± 2	60% ± 38
	100 μM	177% ± 16	21% ± 10	17% ± 5	177% ± 16	21% ± 10
**Farmorubicin**	20 μM	83% ± 33	16% ± 2	0	83% ± 33	16% ± 2
	100 μM	159% ± 9	38% ± 12	30% ± 8	159% ± 9	38% ± 12
